# Immediate Repair of the Soft Parts Following Labor1Read at the 35th annual meeting of the Medical Association of Central New York, held at Syracuse, N. Y., October 21, 1902.

**Published:** 1903-01

**Authors:** Charles E. Congdon

**Affiliations:** Buffalo, N. Y.


### Immediate Repair of the Soft Parts Following Labor.[1]

By CHARLES E. CONGDON, M. D., Buffalo, N. Y.

THE subject of immediate repair of lacerations of the soft parts due to parturition is one that should interest us all. This is a subject upon which at least nearly one-half of gynecology rests; it is a subject so extensive and in its results when unattended, presenting such a variety of phases and often so complex, that it comprises a large percentage of the practice of medicine.

Almost daily I see women with a history of having been in the hands of one or more specialists with little or no benefit, their entire illness dating from a childbirth in which injuries to the parturient canal were unannounced and unattended, or if repaired were unsuccessful.

Many writers are inclined to the belief that the reason these injuries go unannounced is that we are dazzled by the brilliancy and wonderful achievements of modern abdominal surgery; this to a certain extent may be true but popular superstition and prejudice combined with professional jealousy have rendered it unwise and unsafe to inform a patient of her true condition following labor. He is a bold obstetrician who informs his patient that she has a laceration, for if he does, almost as sure as fate, some rival will assert that it was unnecessary and avoidable; therefore in order to popularise the work of immediate repair it becomes necessary for us as a body to point out the necessity or to await the demands of the laity—a day which is not far distant.

*Diagnosis.*—It should be remembered that these injuries occur more frequently internally than externally; that is, they always begin from within, and may be superficial lacerations or they may extend through any or all of the soft parts. I have seen lacerations that opened the peritoneal cavity, that lacerated the uterus beyond the internal os, extending through the broad ligament, through all the soft parts opening up the ischio-rectal space; lacerations anterior into the urethra or on one or both sides of the urethra, extending through the vestibule, even lacerating the hood of the clitoris, through both labia. The more common lacerations are in one or both sulci, and, as I said before, of varying degree.

---

1. Read at the 35th annual meeting of the Medical Association of Central New York, held at Syracuse, N. Y., October 21, 1902.

*Prognosis.*—The prognosis in these cases of immediate repair is good; the hemorrhage, which is quite profuse, ceases instantly upon tying the properly introduced sutures. In my first cases I was very much surprised at the slight amount of hemorrhage, at the lessened amount of lochia and the easy and rapid recovery the patients so treated made. The reason for this is to be attributed to the lessened shock from hemorrhage, the lessened exhaustion which follows the lochial discharge, and last but not least there is no infection area.

*Treatment.*—As regards the prophylaxis it is impossible in many instances to prevent the occurrence of lacerations. I have seen many lacerations and none so extensive as where instruments were not used; on the other hand, I am inclined to believe that instruments in some cases have been the cause and this can only be overcome by their judicious use when the case requires instrumental delivery.

The subject of the prophylaxis of injuries to the parturient canal in justice to itself requires an entire paper, therefore I will dismiss the subject by saying that I do not believe that the mind of man ever has or ever will be able to formulate principles and details so that injuries to the parturient canal, the result of labor, will become a matter of history.

The method which I follow is to put the patient in the lithotomy position, introduce retractors, so as to expose the cervix, grasp it with a tenaculum, and slight traction brings it to the vulva where the sutures may be easily introduced. As soon as this is accomplished the uterus is replaced, the vagina cleansed from blood-clots when it will be noted that the blood which obscures the field of observation disappears, making the repair work on the vulva and vagina very simple; in fact, I believe this to be the key to the successful finding and uniting of these injuries.

Some obstetricians recommend the introduction into the vagina against the cervix of a tampon in order to keep the field of observation from being obscured by the hemorrhage coming from the uterus. This is unnecessary when the lacerated uterine tissues are united.

Now by properly introduced sutures, if the sundered tissues are brought into perfect apposition, primary union will almost invariably be secured; if the repair work has been properly executed the after-treatment is of little moment.

1034 JEFFERSON STREET.

### DISCUSSION.

Dr. A. B. Miller, Syracuse.—The subject is an interesting one, and one which should be of special interest to the general practitioner. Dr. Bannan, who has a wide experience in treating these cases, is here, and I would like to ask the chair to call on her to discuss this paper.

Dr. T. Bannan.—I consider that I am the victim of an unfair advantage in being given the privilege of the floor; also, I deny the ability to discuss this paper as it should be discussed. My experience in obstetrics has been confined, as far as laceration is concerned, to prophylaxis. I do not think any repair can begin to compare with the prevention of laceration. I have never seen, even when they were involved, the extended lacerations as have been mentioned in the paper. Another thing, at the end of twelve, fifteen, eighteen, twenty-four hours of labor a woman is rarely in condition to undergo even the repair suggested. The physician, if he has been attentive, is rarely in condition to give a satisfactory repair. The experience that lacerations are more extensive without forceps than with has not been in my line. The trouble in most cases is laregly a moral one, where the woman becomes impatient, the doctor equally impatient, where forceps are applied before the cervix is ready for it, where the membranes are ruptured before they have completely dilated the cervix, before the peritoneum has begun to relax, the forceps put on and the child extracted, without any attention paid to extraction during the pain. These are the conditions which lead to laceration, and in the vast majority of cases where we know the children are born without any attending physician, it certainly does not seem right in the harmony of things to say that an extensive sewing is necessary after a normal parturition. Not one thing more than another should be emphasised than the conduct of labor in a natural way, to know when it is natural, to know what a natural delivery is, what the prevention is, what the hygiene of pregnancy is. If we understood those things, and if our medical students were taught the power of nature itself, then they will know when to put on instruments and when not to put them on, and we will not have the extended laceration nor the need for the surgical repair which this paper has offered.

Dr. R. M. Moore, Rochester.—I do not agree with the last speaker exactly, because I think we always encounter lacerations of the cervix in some degree in every case of labor. I do not think a woman will have a baby without some laceration of the cervix, and even in small lacerations sometimes there will be an ectropion of the mucous membrane. The laceration itself is a matter of no moment, provided we do not have this ectropion surface. I have been paying some attention to this subject for two years past, and I find that my patients do a great deal better if a recent repair of the cervix at least is made. I do not agree with the writer that an immediate repair is wise. We have a very elongated cervix to deal with and the tissues are very flabby, they do not hold the stitches well, but if a recent repair is made and by that I mean within the first 48 hours, or immediately after 48 hours, we have a sufficient involution occurring, especially of the cervix, so that it makes it a very easy, simple matter and one that is invariably successful, provided we do not have an infection, like gonorrhea. The reader did not speak of one thing that I think in regard to the technique is a necessary one. You can't bring the cervix to the vulva easily unless you empty the bladder absolutely, and although the patient may have urinated, and the nurse will tell you, "why she has just emptied her bladder," you will find if you put in a catheter and make pressure above that there are four or five ounces of urine left behind, and without emptying the bladder completely you cannot bring the uterus to the vulva as you should. I have tried a number of sutures. I have found that the easiest and the most simply applied and the one that acted the best was a simple continuous ball stitch of a very small size. There was another thing I noticed also in this, that you must sew the cervix up very small, you have got to make it almost complete, so that you can just introduce your finger into the cervix after it is through. If you don't do that, when you examine your patient again in a couple of weeks you will find that you have not repaired the cervix completely; you will think that you have done so, but there still remains a slight defect. The patients that have their cervices repaired will have involution go on much more rapidly than those that have not had repair done. They convalesce much more quickly. They get up free from a leucorrheal discharge, free from that pain in the back which so many women have. I have operated upon or have sewed up between forty and fifty cases. I have at first simply sewed those cases that were primiparous. Of late I have been making the operation on multiparas as well. That is, those whom I had examined and found with an old laceration they were suffering from, I go right on and make the operation. I think it is much easier to do it. That saves your convalescent from the confinement, the trouble of a second operation. I agree with the reader of the paper that these things should be taken up, not immediately, but within forty-eight hours.

Dr. A. B. Miller.—I feel it would be unfortunate to leave the impression with this body of medical practitioners that we are in sympathy with doing repair on the soft parts immediately, especially of those of the cervix. I was not under the impression that the paper dealt with the lacerations especially of the cervix. We have here a body of fibrous tissue, in whch in the course of delivery the cervix is completely taken up. We have a complete obliteration of the cervix. We know as a positive fact that involution cannot take place under three or four months, that the cervix will be scarcely perceptible for a week or ten days.

I believe with the last speaker that lacerations do take place in the majority of cases, but nature repairs or takes care of the majority of them. All the anterior and posterior lacerations are repaired by nature. We do occasionally find the unilateral or bilateral laceration that requires an operation. I know it is the teaching of special men in this department that the time to do an operation in the fibrous tissue is not immediately following parturition. The paper deals, or at least I supposed it dealt, with lacerations of the soft parts, and laceration of the soft parts as I understand means the pelvic floor and not the perineum. When it comes to injuries of soft parts, as stated in the paper, I am pleased to say I have never seen anything of the nature of that spoken of by the author, with one exception, and that should have been discussed this morning when the paper was read on eclampsia. I believe in immediate repair of the soft parts of the perineum and extending up into the vagina; I do not believe in doing repairs to the fibrous structures.

Dr. J. P. Creveling.—There may be some men here like myself located in rural districts where we have to get along the best we can without trained nurses or assistants. We get a perineum laceration and we cannot help ourselves at times. Somehow, I believe the best thing under these circumstances is to tell the people and then, without any consideration of professional jealousy, proceed to sew up that rent just as well as we can and just as soon as we can.

President Beahan.—I have taken considerable interest in this subject in the past year, and think I will join the Rochester contingent. I read a paper last year before the Medical Society of the State of New York at the October meeting, which Dr. Charles Judd, of Brooklyn, discussed, and he was rather severe on the position assumed, namely—that these injuries should be repaired immediately, but after the meeting I had a conversation with him, and he was very glad to know what was being done in this and other regions in regard to this matter. In the various comments in the several journals that I happened to read, the position assumed by that paper, which was of immediate repair, was favorably spoken about. I consider that this paper is in the right spirit, and that the immediate repair of all the parts pertaining to parturition is worth considering, not only of the cervix but also the perineum, and that the woman after confinement is entitled to be placed in the same condition she was before her confinement, that the laceration should not continue for an indefinite length of time before repair, if it can be restored, as Dr. Moore has indicated, and as has been developed in Rochester. The subject is well worth further consideration and your interest. Within a year I have seen two instances of attempted suicide in women who have had lacerations, both of the cervix and of the perineum, and it seems probable that the indirect cause of this condition of the mind was due to the old laceration. In each of these instances, too, attempts had been made to repair the lacerations so far as the perineum was concerned, and in one of them with regard to the cervix. I believe that after involution has partially been effected by the influence of douches, by the administration of ergot, and then by putting the patient in the best possible position, as Dr. Congdon has indicated, and in a good light, let everything be properly cared for, and let the patient be put upon her feet in good condition at the end of two or three weeks, rather than wait until an indefinite time, when somebody will discover and repair the rents. A woman I think is entitled to immediate attention, and not at a remote time.

Dr. C. E. Congdon.—I am very much pleased that this paper brought out such a discussion. I wish to say, in justice to myself, that these patients are usually brought to the hospital for repair. I may say also that I have confined several cases and I have examined them, and I have had men present who never saw a laceration, but they did not walk out of that room without seeing one, and I frequently give them the privilege of observing the patient from the beginning of the pain to the finish; and they will generally find extensive lacerations of the cervix. I wish to ask the doctor who very kindly opened the discussion if she ever examined a patient herself for lacerations?

Dr. Bannan.—Yes.

Dr. Congdon.—Then I will refer you to Matthew, VII. Chapter and 7th verse. As to prevention, naturally prevention, is better than repair. I do not believe it is possible for any one to restore anything to its original perfection. In regard to lacerations of the cervix, Dr. Moore is right when he says you cannot bring the entire cervix to the vulva, but you can bring the cervix to the vulva, as I stated in my paper, with properly introduced retractors on both sides; you will then have very little difficulty in bringing the surfaces together; and I was very glad he brought out the point which I failed to refer to in the paper, and that is, how extremely small the cervix is when the surfaces are coaptated; you may just get your finger in it. Then by introducing the silkworm-gut sutures one after another, carefully adjusted, the parts can be made to approach their natural state. This subject was discussed by the American Association of Obstetricians and Gynecologists in Washington some time ago, and it was the consensus of opinion that these injuries should be repaired immediately, but in cases where the surroundings were filthy and unsanitary there was believed to be great danger of infection. This paper was by Dr. Ill.

The point was touched upon as regards the appropriate time for repair of the cervix, and Dr. Moore suggested forty-eight hours. Forty-eight hours might be a good time, but I believe the general consensus of opinion is that within twenty-four hours constitutes immediate repair. Dr. Miller suggested trusting these repairs to nature. I don't know but that it may be a wise plan, but I think for a number of years we have trusted these things to nature until nearly every woman who has borne children will be found, upon examination with the speculum, to have a large hypertrophic cervix, with induration, and an ectropion surface, with a foul smelling purulent discharge from it that is exhausting her, until she becomes so pale and anemic that she is not capable of taking care of her work or her family, and life is unbearable; and those cases I want to say right here nature will never cure. They are beyond it; it is too late. Nothing but a radical operation will be of the slightest benefit.

As regards the vagina, some of the parts are brought together to prevent infection, but as regards the perineum, that naturally is very easy to repair at the time of rupture. Dr. Moore mentioned repairing old lacerations at the same time. This, too, is very simple and very easy, but the fascia and muscle should be brought together. One word more, and that is, when you have a cervix that is lacerated, perfect muscular contraction cannot take place. The cervix is opened up, and you know that in the lining of the cervix various glands are lodged there. They become irritated and congested, and care should be taken to excise them in old cervical tears. Unless this is done these surfaces become a nidus for cancerous disease.

